# Susan Britton Wills Unit, Bristol General Hospital, Multidisciplinary, General Hospital and Community Psychiatry

**Published:** 1989-02

**Authors:** Glin Bennet, David Cook, Jan Smith

**Affiliations:** Consultant Senior Lecturer; Consultant Senior Lecturer; Lecturer, Department of Mental Health, University of Bristol

## Abstract

The Susan Britton Wills Unit at the Bristol General Hospital is a small and busy multidisciplinary psychiatric unit, providing a wide range of in-patient, out-patient and day-patient services for the residents of Bristol Health District.


### Susan Britton Wills Unit, Bristol General Hospital: multidisciplinary, general hospital and community psychiatry

**Glin Bennet MA, MD, FRCS, FRCPsych**
Consultant Senior Lecturer

**David Cook MA, BM, BCh, MRCPsych**
Consultant Senior Lecturer

**Jan Smith MB, ChB, MRCPsych**
Lecturer, Department of Mental Health, University of Bristol

### SUMMARY
The Susan Britton Wills Unit at the Bristol General Hospital is a small and busy multidisciplinary psychiatric unit, providing a wide range of in-patient, out-patient and day-patient services for the residents of Bristol Health District.

### INTRODUCTION
Setting up a psychiatric unit in the Bristol General Hospital meant taking our services closer to where the residents of south Bristol actually live and work. This has made possible a flexible kind of care for people in crisis. They come to us from all the social agencies and they can refer themselves through the Accident Department of the Bristol Royal Infirmary.

We offer consultations in the constituent hospitals of the group, out-patient sessions at the BRI, day care and in-patient care at the BGH, and a variety of groups run outside hospital premises.

The Unit was conceived in 1969 as a way of amalgamating the clinical parts of the Professorial Department of Mental Health—previously split between the Bristol Royal Infirmary and the Homeopathic Hospital—into a larger and better placed clinical department. The building offered was the Susan Britton Wills sisters' home at the BGH. The location was ideal for promoting the Department of Mental Health's policy of bringing services out to the people, even though the concept of community care was unfashionable in that era when resources were being concentrated in large hospital complexes.

The building we were given was far too small, but we had a firm promise that a large day hospital would be provided in the very near future so that we could develop the community services we felt were needed. (That day hospital plan was killed off as a result of one of the periodical NHS reorganisations. There was also a plan for a 120-bedded inpatient psychiatric unit on the BRI site, but that, too, was dropped). However, the cramped conditions did have one unexpected benefit: we all had to interact. Staff could not retreat to their private departments as is liable to happen in large organisations. We were stacked on top of one another, we had to make the best of it and did.

### The SBW Unit
There are three floors to the unit, and the first two contain the 26 in-patient beds. On the ground floor are the beds for the acute admissions—a single-bedded observation room, a single-bedded and a four-bedded room, and a ten-bedded (usually female) ward. They are furnished with divan beds and fairly domestic looking furniture. On the first floor are two three-bedded rooms and one four-bedded room for people who require a minimum of supervision, there is an art therapy room, the Social Work department and interview rooms. On the top floor is the Occupational Therapy department, dining area, conference room and video suite. The Day Hospital functions on the top floor, although it does not actually have any defined area except one small office. It is a day hospital more in terms of the work it does, inside and outside hospital premises, than the space it occupies.

The ready accessibility of the Unit is one of its great advantages. There are 14 bus routes within four minutes walk, and another 16 and Temple Meads station within ten minutes walk.

### Staff and work
The staff in the SBW Unit are in medicine, nursing, social work, occupational therapy, art therapy and psychology. Patients each have a primary therapist who is responsible for guiding treatment and who may be from any of the disciplines. Each patient also has a doctor who may be primary therapist or merely acting in a kind of general practitioner role during attendance at or in hospital. The main approach is psychotherapeutic, that is we try to work on the psychological origins of people's problems and/or their resolution by psychological means. Once the main problem has been identified, or at any rate the area on which we have agreed with the patient to work, we design a programme of activities and psychotherapeutic sessions to meet the individual's needs. These needs are worked out by discussion with members of the team on entering out-patient, day-patient or in-patient care, and as needs change there can be an easy transition to a different type of care because either they will remain in the same building or be with the same staff or both. Wherever possible we work with partners and families.

Having said that, we are part of the psychiatric service for Bristol and so are available to everybody in need of psychiatric care. Many are admitted in crisis, having reached the end of their tether, and require respite. Some are depressed, feeling hopeless about their lives, some are hyper-excited or otherwise acutely disturbed and require to be contained in a hospital. Occasionally patients are very violent or sexually disinhibited, in which case they can go to the new acute admission unit at Barrow Hospital.

Drugs are used to help people to relax (in addition to relaxation exercises), to relieve distress, to help lift a depression, and for sedation when that is required. ECT is also used occasionally, but much less often than a decade ago, as the following comparison shows (complete figures for 1986 are not available).

*Total numbers of treatments given (not numbers of patients treated)*

| | |
|---|---|
| 1972—363 | 1982—72 |
| 1973—330 | 1983—115 |
| 1974—306 | 1984—89 |
| 1975—326 | 1985—141 |

The turn-over in the SBW Unit is high.

| | *number of admissions* | *average length of stay (days)* | *% bed occupancy* |
|---|---|---|---|
| 1980 | 236 | 25.6 | 66.6 |
| 1985 | 229 | 37 | 88 |
| 1986 | 283 | 31.1 | 90.5 |

The median length of stay would have been preferred to the average as it is less distorted by patients remaining in the Unit for extended periods. Nearly always there are one or two patients who have been in the Unit for a year or more, and recently a girl, now 19, has been an in-patient for over two years. There are of course no long stay psychiatric beds in Bristol Health District, nor any in-patient adolescent facilities. The median length of stay is a difficult calculation for the Regional computer, but it was calculated for 1986 as 11 days, based on a 97.5% sample of 1986 admissions. A low median or average length of stay is only meaningful if accompanied by readmission rates, and on the same sample 11.4% were readmitted once (i.e. 2 admissions altogether), 2.2% twice, and 2.2% three or more times.

The policy of the SBW Unit is to admit in crisis, and to keep patients in hospital only for the duration of the crisis, and then continue care at the Day Hospital or as out-patients. During in-patient treatment contact is maintained with families, friends and the community as far as is practicable. It is easy for most in-patients to travel home and to spend days and nights at home as they progress, to keep in touch with life and activities in Bristol, and where desirable to go out to work from the Unit. In a significant proportion this positive progress is impossible because the people have nowhere to go or relatives are unable to care for them. Thus they hang around in an acute unit watching others come and go, and so they are liable to feel unwanted and in the way.

In addition to a heavy load there is active teaching at the SBW Unit. There are medical students, trainee general practitioners, trainee psychiatrists, student nurses from the BRI and Barrow Hospital, social work, psychology and occupational therapy students. The family therapy team is committed to training as well as to therapy.

### Day Hospital

The main function of the Day Hospital is to care for those people who are unable to live ordinary lives on account of their problems, yet do not need 24-hour-a-day support. There are usually 12 to 15 day patients attending at any one time (and about 50 on the books), and the space available has to be shared to meet the occupational-therapy needs of the 25 in-patients.

Increasingly people are referred to the Day Hospital for specific purposes such as psychotherapy, general assessment of function, or for some of the group activities available: the list is formidable. There are therapeutic groups for anxiety management, communication and social skills, and women's issues; and others using music, drama, art, pottery, carpentry, massage and relaxation. There are groups offering outings, swimming, cookery and advice about healthy eating, a support group for elderly people, and a group where people receiving depot medication can be supervised and meet one another. These groups take place in a variety of settings in south Bristol.

### Psychiatric clinics at health centres

From time to time various psychiatric consultants from the SBW Unit have conducted clinics at health centres, and at present Dr David Cook's team is running one at Montpelier Health Centre which is very busy and generates a great deal of crisis work.

### Conclusion

The centrally located SBW Unit, staffed by energetic and imaginative people from different disciplines, is offering a unique service in south Bristol. The flexible approach combining in-patient, out-patient and day-patient care is proving highly successful in meeting the ever-increasing demand for services within limited budgets.

